# Treatment patterns and cost of exacerbations in patients with chronic obstructive pulmonary disease using multiple inhaler triple therapy in South Korea

**DOI:** 10.1186/s12931-022-02136-0

**Published:** 2022-09-05

**Authors:** Chang-Hoon Lee, Mi-Sook Kim, See-Hwee Yeo, Chin-Kook Rhee, Heung-Woo Park, Bo-Ram Yang, Joongyub Lee, Eun-Yeong Cho, Xiaomeng Xu, Aldo Amador Navarro Rojas, Sumitra Shantakumar, Dominique Milea, Nam-Kyong Choi

**Affiliations:** 1 Division of Pulmonary and Critical Care Medicine, Department of Internal Medicine, Seoul National University Hospital, Seoul, South Korea; 2 Medical Research Collaborating Center, Seoul National University Hospital, Seoul, South Korea; 3 Value Evidence & Outcomes, GlaxoSmithKline, Singapore, Singapore; 4 Division of Pulmonary and Critical Care Medicine, Department of Internal Medicine, Seoul St. Mary’s Hospital, College of Medicine, The Catholic University of Korea, Seoul, South Korea; 5 Department of Internal Medicine, Seoul National University, Seoul, South Korea; 6 Institute of Allergy and Clinical Immunology, Seoul National University Medical Research Center, Seoul, South Korea; 7 Department of Internal Medicine, Seoul National University College of Medicine, Seoul, South Korea; 8 College of Pharmacy, Chungnam National University, Daejeon, South Korea; 9 Department of Preventive Medicine, Seoul National University College of Medicine, Seoul, South Korea; 10 MA Respiratory Department, GlaxoSmithKline, Seoul, South Korea; 11 Department of Health Convergence, College of Science and Industry Convergence, Ewha Womans University, Seoul, South Korea

**Keywords:** Adherence, Claims database, Chronic obstructive pulmonary disease, Exacerbations, Persistence, Real-world, South Korea, Treatment patterns, Triple therapy

## Abstract

**Background:**

Multiple inhaler triple therapy (MITT), comprising inhaled corticosteroids (ICS), long-acting beta-agonists (LABA), and long-acting muscarinic antagonists (LAMA), has been used as an escalation treatment for patients with chronic obstructive pulmonary disease (COPD). However, real-world use of MITT has not been investigated in Asia, including South Korea. This study reports baseline characteristics of patients with COPD initiated on MITT in South Korea, and their treatment patterns. Healthcare resource utilization (HRU) and costs associated with COPD exacerbations following MITT initiation were also assessed.

**Methods:**

This was a retrospective cohort study using the South Korea National Health Insurance database (2014–2018). Included patients were ≥ 40 years, had a COPD diagnosis, were newly initiated on MITT and had ≥ 12 months’ data both before (baseline) and after index date (the first day with overlapping supply of all MITT components). Treatment immediately before initiation and immediately following discontinuation of MITT were identified, and proportion of days covered (PDC) by MITT was calculated. HRU and costs (per person per year [PPPY]) associated with exacerbations were identified following MITT initiation; costs were calculated using the average 2020 exchange rate (0.0008 USD/KRW).

**Results:**

Among 37,400 patients, the mean age was 69 (SD 10) years and 73% were males; 56% had ≥ 1 COPD exacerbation during the baseline period, with a mean of 2 (SD 5) events/year. ICS/LABA was the most frequent regimen prescribed immediately before initiation (37%) and immediately following discontinuation (41% of 34,264 patients) of MITT. At 3, 6, and 12 months from treatment initiation, mean PDC was 81%, 63% and 49%, respectively; median treatment duration was 102 days. The mean (95% confidence interval [CI]) number of total visits for severe COPD exacerbations was 0.77 PPPY (0.75–0.78); mean PPPY total healthcare costs were 2093 USD.

**Conclusions:**

Patients with COPD in South Korea experienced frequent exacerbations prior to MITT, and PDC by MITT was low. Patients may benefit from early optimization of COPD therapy, and greater emphasis on adherence to inhaled COPD therapy. Severe exacerbations were found to incur substantial costs; treatment alternatives that can reduce the rate of severe exacerbations are likely to minimize healthcare costs.

**Supplementary Information:**

The online version contains supplementary material available at 10.1186/s12931-022-02136-0.

## Background

Chronic obstructive pulmonary disease (COPD) is a common progressive inflammatory disease that causes irreversible airflow limitation. Patients with COPD experience shortness of breath, wheezing, and exercise intolerance [1], resulting in an impaired ability to conduct activities of daily living and a lower quality of life [2]. COPD imposes a substantial mortality burden and is predicted to become the fourth leading cause of death worldwide by 2030 [3]. In South Korea, the annual prevalence of COPD among adults aged ≥ 40 years rose from 12% in 2010 to 16% in 2012, with higher prevalence rates reported in patients who are males and of older age, as well as in those with higher smoking intensity and lower body mass index [4]. As of 2010, in South Korea, chronic lower respiratory diseases including COPD were the seventh leading cause of death in the overall population, and the fifth leading cause of death among those aged ≥ 80 years [5]. COPD is also associated with a substantial economic burden, where 20-year direct medical costs in the United States (US) were estimated to be $800 billion US dollars (USD) between 2019 and 2038 [6]. In South Korea, the medical cost per patient with COPD was 1183–3744 USD per year [7, 8], with a total annual average of 51 days spent in hospital or outpatient care [8].

A key treatment goal in the management of COPD is to reduce symptoms and prevent future exacerbations [9]. For patients who continue to experience frequent exacerbations and persistent breathlessness while on inhaled corticosteroids (ICS) and long-acting beta-agonists (LABA), or LABA and long-acting muscarinic antagonists (LAMA), the Global Initiative for Chronic Obstructive Lung Disease (GOLD) 2017 Report suggests that treatment may be escalated to triple therapy–this suggestion was maintained in the GOLD 2020 Report that was released after this study was completed [9, 10]. Triple therapy comprises simultaneous receipt of ICS, LABA and LAMA, in combinations of ICS/LABA + LAMA, ICS + LABA/LAMA, or ICS + LABA + LAMA (i.e., multiple inhaler triple therapy [MITT]); ICS/LABA/LAMA as a single inhaler triple therapy (SITT) was not available in South Korea at the time of this study. Step-up to triple therapy can improve lung function and patient-reported outcomes [11], and prevent exacerbations [9].

Use of MITT has been reported in previous real-world studies from Western regions. A study in the US reported that a quarter of patients with COPD receiving at least one LAMA, LABA, ICS or phosphodiesterase-4 inhibitor progressed to MITT within 2 years of COPD diagnosis [12]. Similarly, a study in the United Kingdom (UK) reported that 32% of patients with COPD received MITT, of whom 25% were prescribed MITT within 1 year of diagnosis [13]. The median time from COPD diagnosis to MITT ranged from 17 months (Australia) to 43 months (UK) [14]. However, real-world use of MITT in Asia, including South Korea, has not been investigated. Although the healthcare use and economic burden associated with COPD in South Korea has previously been reported, patients in these studies were most commonly treated with oral medications [7, 8]. A real-world study in South Korea investigating the use of inhaled COPD treatment, including MITT, is therefore needed.

This study aimed to assess the use of MITT by South Korean patients with COPD in a real-world setting, in order to understand COPD control and medication use, and to inform treatment choices. Understanding the real-world use of MITT in South Korea may also facilitate comparisons with future changes in treatment practice, such as the introduction of SITT, which was not available at the time this study was conducted. Here, we report baseline demographics and clinical characteristics of patients who initiated MITT, treatment patterns prior to initiation, during, and after discontinuation of MITT, adherence to and persistence with treatment following initiation of MITT, and healthcare resource utilization (HRU) and costs associated with COPD exacerbation events.

## Methods

### Study design and data source

This was a retrospective cohort study using the South Korea National Health Insurance (NHI) Database from January 2014–December 2018. Claims data from the NHI Database cover the entire South Korean population across all medical facilities, including primary, secondary, and tertiary healthcare institutions. The NHI Database includes information on patient demographics, diagnoses, prescribed drugs, procedures for medical services, site of care, costs, and medical utilization for claims made.

### Study population

**Fig. 1:**
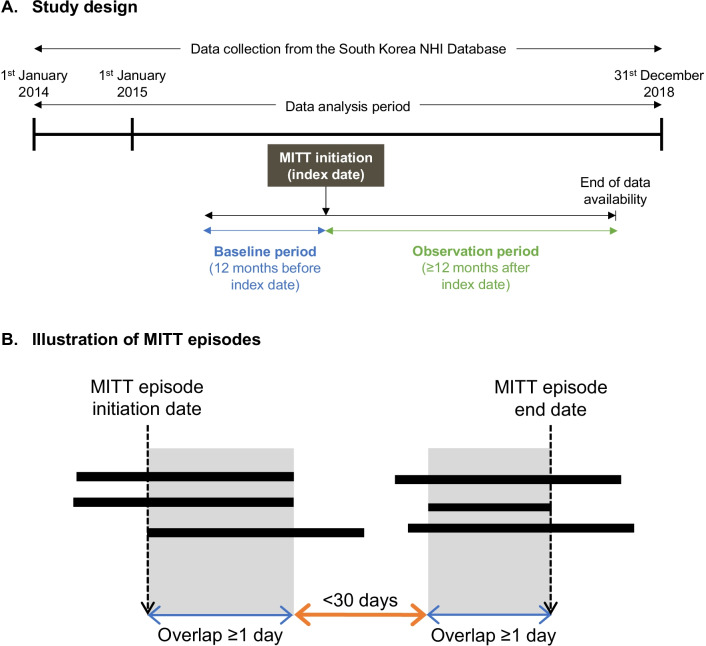
Study methodology. **A** Study design. Index date was the date of MITT initiation, defined as the first day with overlapping supply of all MITT components. The baseline period comprised the 12 month-period prior to index date, and the observation period comprised the ≥ 12-month period from index date until the end of data availability (≥ 12 months, until the cut-off for data availability [31 December 2018]). MITT: multiple inhaler triple therapy; NHI: National Health Insurance. **B** Illustration of MITT episodes. Each black bar represents a dispensing for a component inhaler of MITT. Each region shaded in grey represents an episode of MITT in which there was continuous overlap of ≥ 1 day for all three MITT components; MITT episodes were considered to be one extended MITT episode if the MITT episodes were < 30 days apart (grace period). MITT: multiple inhaler triple therapy

Eligible patients were ≥ 40 years with a diagnosis of COPD (International Classification of Diseases, 10th Revision, Clinical Modification [ICD-10-CM] J43–J44); ≥ 2 independent records were required in an outpatient setting, or ≥ 1 record was required in an inpatient or emergency room (ER) setting. Eligible patients were newly initiated on MITT with ≥ 1 filled prescription of any MITT component, and had ≥ 12 months of data both before (defined as the baseline period) and after the index date. The index date was the date of MITT initiation, which was defined as the first day with overlapping supply of all MITT components. The observation period comprised the time from index date until the end of data availability (≥ 12 months, until the cut-off for data availability [31 December 2018]) (Fig. 1A).

Patients were excluded if they did not meet the inclusion criteria described above, i.e., patients who were < 40 years, who did not have a sufficiently long baseline or observation period (< 12 months from their index date or prior to data cut-off at December 2018), and/or who did not have the relevant COPD-related claims (≤ 1 COPD-associated outpatient claim and no inpatient or ER claims at the index date or in the year prior to the index date). Patients were also excluded if they had a baseline diagnosis for any malignancy (including lung cancer and respiratory metastasis), scoliosis/kyphosis, chronic pleurisy, or mycobacterial disease (including tuberculosis and non-tuberculosis mycobacterial pulmonary infections). Patients with these conditions were excluded as they may present with similar symptoms to COPD, such as dyspnea and frequent coughing. HRU and associated costs may be confounded by these other conditions, and thus may not be an accurate reflection of COPD alone.

### MITT exposure

MITT use was defined as concomitant use of two different inhalers in the form of ICS/LABA + LAMA or ICS + LABA/LAMA, or of three different inhalers in the form of ICS + LABA + LAMA. An episode of MITT comprised the period in which there was continuous overlap of ≥ 1 day for all three MITT components; episodes that had a gap of < 30 days (grace period) were considered to be one extended episode, wherein patients were considered to be on MITT during the entire period (Fig. 1B). This grace period was added to account for patients’ medication supply lasting longer than the days’ supply due to usage or compliance issues, and is commonly applied in studies evaluating medication persistence—a median grace period of 30 days has been reported in published studies [15].

### Outcomes

Baseline characteristics and demographics such as age and sex were assessed at the index date. Clinical characteristics such as COPD exacerbation history and comorbidities were assessed during the 12-month baseline period. Elixhauser comorbidities were identified [16], and the mean Charlson Comorbidity Index score was calculated [17]. Component conditions and the respective ICD-10-CM codes are listed in Additional file 1: Tables S1 and S2.

Consistent with definitions from the GOLD 2017 Report, a moderate COPD exacerbation was defined as an outpatient visit with a primary COPD diagnosis and ≥ 1 dispensing of a systemic corticosteroid or antibiotic within seven days, and a severe COPD exacerbation was defined as an inpatient hospital stay or ER visit with a primary COPD diagnosis [9]. If a moderate and severe COPD exacerbation event were identified within 14 days of each other, this was counted as a single severe COPD exacerbation event based on the rationale that the initial moderate COPD exacerbation could have worsened to become a severe COPD exacerbation. HRU associated with outpatient visits, ER visits, and hospitalizations was assessed during the 12-month baseline period.

Treatment patterns prior to initiation, during, and following MITT use were assessed using pharmacy dispensing data. Treatment patterns immediately prior to initiation and immediately following discontinuation of the first episode of MITT were assessed; treatment had to be dispensed within 90 days of the first episode of MITT.

Proportion of days covered (PDC), adherence, and persistence were also calculated to assess real-world utilization of MITT. PDC was defined as the number of days on MITT therapy over a fixed time interval, expressed as a percentage. Calculation of PDC did not count days of hospitalization; this was deemed to have minimal impact on underestimation of adherence in a setting with a mean hospitalization duration of < 28 days per year, such as in South Korea [8, 18]. Adherence was defined as PDC by MITT ≥ 80%, which was in line with definitions from the International Society for Pharmacoeconomics and Outcomes Research checklist for medication compliance and persistence studies conducted using retrospective databases [19]. Persistence was defined as the percentage of patients with continuous MITT use with a gap of < 30 days between any component. PDC, adherence, and persistence were assessed at 3, 6, 12, 18 and 24-month intervals after MITT initiation. Sensitivity analyses for PDC and adherence were conducted by broadening the definition of MITT from concomitant use of all MITT components to use of any MITT component (Additional file 1: Fig. 1). Sensitivity analyses for persistence were conducted by broadening the 30-day grace period to a 90-day grace period.

HRU and costs associated with moderate and severe exacerbations were assessed after MITT initiation. HRU included COPD-related outpatient and ER visits, and hospitalization and length of stay; costs included pharmacy costs, and costs associated with COPD exacerbation-related outpatient visits, ER visits and hospitalizations. HRU and costs were expressed in per person per year (PPPY) terms; costs were estimated by multiplying per person per month (PPPM) values by 12. Costs in South Korean Won (KRW; ₩) were indexed to 2016 and converted to USD using the average 2020 exchange rate of 0.0008 USD/KRW. Costs were calculated as the sum of the payer’s amount and patients’ out of pocket amounts; the cost of medication during hospitalization was included in the hospitalization cost.

### Statistical analysis

Data were reported descriptively, and no statistical tests were performed. Descriptive statistics for baseline characteristics, such as patient baseline demographics, clinical characteristics, and HRU, were reported as mean (SD) for continuous variables, and percentages for categorical variables. Outcomes relating to treatment patterns surrounding MITT use were similarly reported as mean (SD) for continuous variables, and percentages for categorical variables. PDC, adherence and persistence were expressed as percentages. HRU and costs associated with COPD exacerbations were reported as mean (SD) or mean (95% confidence interval [CI]) for continuous variables, and percentages for categorical variables.

## Results

### Study population, baseline clinical characteristics, and HRU

**Fig. 2:**
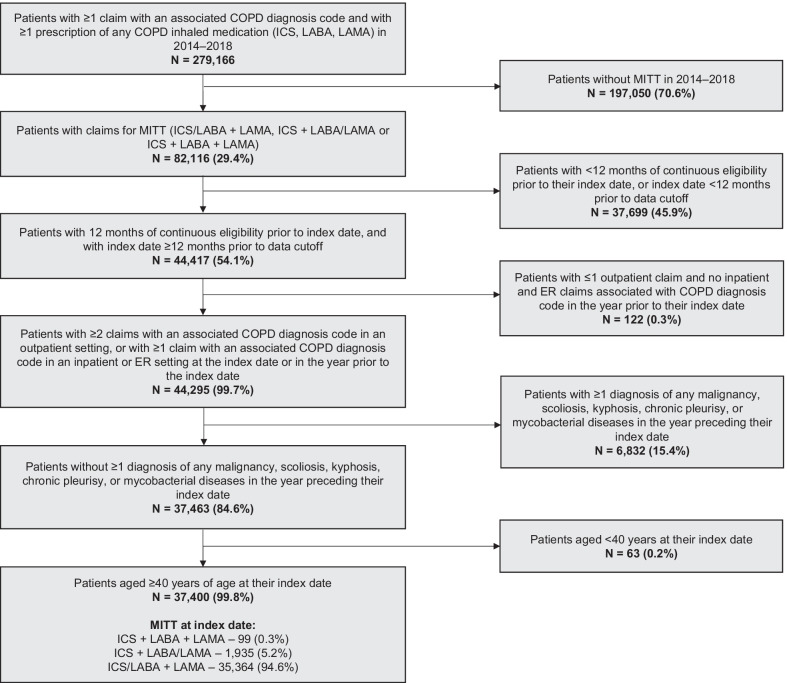
Patient flow and eligibility criteria. Index date was defined as the first overlapping day of supply with all three COPD medications of the MITT regimen; patients’ index date was ≥ 12 months prior to data cutoff. COPD: chronic obstructive pulmonary disease; ER: emergency room; ICS: inhaled corticosteroid; LABA: long-acting beta agonist; LAMA: long-acting muscarinic antagonist; MITT: multiple inhaler triple therapy

Of the initial 279,166 patients identified from the database between January 2014 and December 2018, 29% of patients received MITT during this period. After excluding patients who did not meet the inclusion criteria surrounding age, duration of data availability, COPD-related claims, and comorbidities, 37,400 patients were eligible for inclusion (Fig. 2). Among the eligible patients, mean age was 69 (SD 10) years and 73% were males. Over half (56%) of the patients had ≥ 1 COPD exacerbation during the 12-month baseline period, with an average of 2 exacerbations per year; this comprised patients who experienced ≥ 1 moderate and severe exacerbation (40% and 30%, respectively). They also had a Charlson Comorbidity Index of 3 (SD 2); most common comorbidities were uncomplicated hypertension (56%) and hyperlipidemia (44%) (Table 1). During the 12-month baseline period, patients had 0.5 (SD 1.2) and 0.2 (SD 0.6) COPD-related hospitalization and ER visits per year, respectively (Table 1).

**Table 1:** Baseline demographics, clinical characteristics and HRU (N = 37,400)

Baseline demographics and clinical characteristics	
Age (years), mean (SD)	68.8 (10.4)
Age (years) categories, n (%)	
40–49	1734 (4.6)
50–59	5659 (15.1)
60–69	10,732 (28.7)
70–79	13,506 (36.1)
80+	5769 (15.4)
Male, n (%)	27,184 (72.7)
Exacerbation history, n (%)	21,070 (56.3)
Moderate^a^	15,129 (40.5)
Severe^a^	11,126 (29.8)
Number of exacerbations, mean (SD)	2.0 (4.6)
Moderate	1.5 (4.2)
Severe	0.5 (1.3)
Charlson Comorbidity Index^b^, mean (SD)	3.0 (2.0)
Elixhauser comorbidities^c^, n (%)	
Cardiac arrhythmia	4770 (12.8)
Congestive heart failure	6533 (17.5)
Depression	6571 (17.6)
Diabetes with chronic complications	5785 (15.5)
Diabetes without chronic complications	10,961 (29.3)
Fluid and electrolyte disorders	4234 (11.3)
Hypertension, uncomplicated	21,073 (56.3)
Hypertension, complicated	3759 (10.1)
Liver disease	11,501 (30.8)
Peptic ulcer disease	10,394 (27.8)
Peripheral vascular disease	7411 (19.8)
Additional comorbidities	
Hyperlipidemia	16,377 (43.8)

Baseline HRU	
Number of outpatient visits, mean (SD)	
COPD-related	4.1 (7.4)
All-cause	33.1 (29.4)
Number of ER visits, mean (SD)	
COPD-related	0.2 (0.6)
All-cause	0.5 (1.3)
Number of hospitalization visits, mean (SD)	
COPD-related	0.5 (1.2)
All-cause	2.1 (5.2)
Duration of hospitalization days, mean (SD)	
COPD-related	10.9 (9.1)
All-cause	6.7 (8.5)

COPD: chronic obstructive pulmonary disease; ER: emergency room; ICD-10-CM: International Classification of Disease, 10^th^ Edition, Clinical Modification; HRU: healthcare resource utilization; SD: standard deviation

^a^A moderate COPD exacerbation was defined as an outpatient visit with a COPD diagnosis and ≥ 1 dispensing for a systemic corticosteroid or antibiotic within 7 days, and a severe COPD exacerbation was defined as an inpatient hospital stay or ER visit with a COPD diagnosis; if a moderate COPD exacerbation event and a severe COPD exacerbation overlapped, or were identified within 14 days of each other, only the severe COPD exacerbation event was counted. ^b^Charlson Comorbidity Index scores were calculated based on weights assigned to specific comorbidities. ^c^Only Elixhauser comorbidities that were present in > 10% of the population were reported. ICD-10-CM codes for the component comorbidities of the Charlson Comorbidity Index and Elixhauser comorbidities are presented in Additional file 1: Tables S1 and S2 respectively. Asthma was not included in this study, due to the known poor reliability of asthma diagnosis codes as a reflection of true asthma diagnoses in South Korea

### Treatment patterns prior to, during, and following MITT

**Fig. 3:**
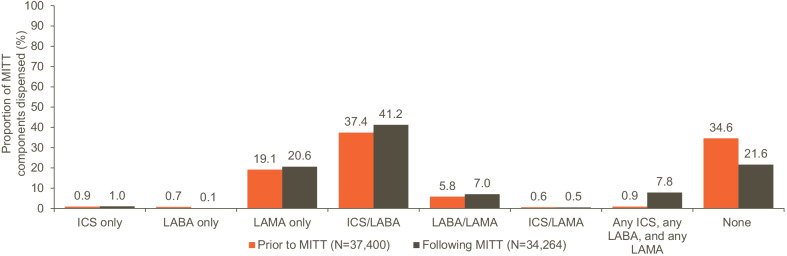
MITT treatment components dispensed immediately prior to initiation and following discontinuation of MITT. MITT was defined as concomitant use of two different inhalers in the form of ICS/LABA + LAMA or ICS + LABA/LAMA, or of three different inhalers in the form of ICS + LAMA + LABA. This figure includes medication with days' supply immediately prior to and immediately following MITT, and within 90 days of the first episode of MITT; results were based on pharmacy dispensing data. Patients may have been treated with more than one medication within a class. ICS: inhaled corticosteroids; LABA: long-acting beta agonist; LAMA: long-acting muscarinic antagonist; MITT: multiple inhaler triple therapy

ICS/LABA was the most frequent regimen dispensed immediately before initiation (37%), followed by no maintenance treatment comprising any of the MITT components (35%), and LAMA only (19%) (Fig. 3). At index date, ICS/LABA + LAMA was the most common MITT regimen (95%), followed by ICS + LABA/LAMA (5%) (Table 2). ICS/LABA was the most frequent regimen dispensed immediately following discontinuation of MITT (41%), followed by no maintenance treatment comprising any of the MITT components (22%), and LAMA only (21%) (Fig. 3).

**Table 2:** Treatment patterns during the first MITT episode (N = 37,400)

Characteristics of first MITT	
Observation period, days, mean (SD) [median (IQR)]	960.5 (314.5) [980 (687, 1240)]
MITT at index date	
ICS + LABA + LAMA, n (%)	99 (0.3)
Duration, days, mean (SD)	223.0 (310.8)
Discontinuation, n (%)	96 (97.0)
ICS + LABA/LAMA, n (%)	1935 (5.2)
Duration, days, mean (SD)	240.6 (250.0)
Discontinuation, n (%)	1697 (87.7)
ICS/LABA + LAMA, n (%)	35,364 (94.6)
Duration, days, mean (SD)	220.8 (267.8)
Discontinuation, n (%)	32,469 (91.8)

ICS: inhaled corticosteroids; IQR: interquartile range; LABA: long-acting beta-agonist; LAMA: long-acting muscarinic antagonist; MITT: multiple inhaler triple therapy; SD: standard deviation

MITT was defined as concomitant use of two different inhalers in the form of ICS/LABA + LAMA or ICS + LABA/LAMA, or of three different inhalers in the form of ICS + LAMA + LABA. The observation period comprised the time from index date until end of data availability

### PDC, adherence to and persistence with MITT

**Fig. 4:**
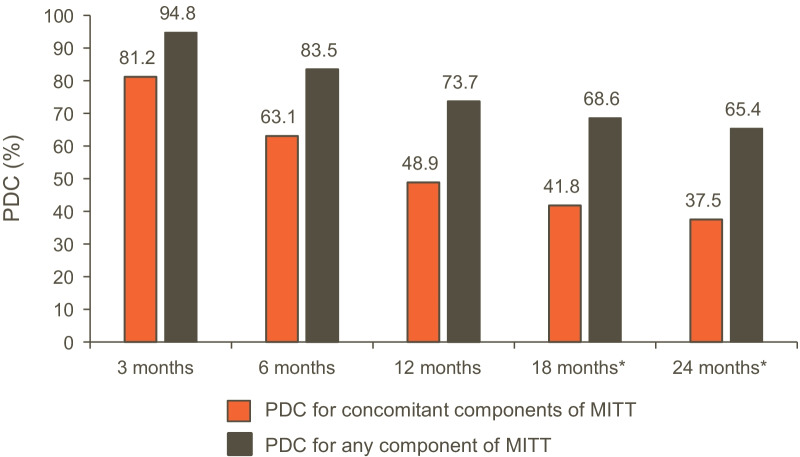
PDC by MITT (N = 37,400). *Total population numbers were 32,743 at 18 months and 26,732 at 24 months. PDC was calculated by dividing the days on therapy for concomitant components or any component of MITT by a fixed time interval, multiplied by 100%. The denominator was 91 days for 3 months, 183 days for 6 months, 365 days for 12 months, 548 days for 18 months, and 730 days for 24 months. MITT: multiple inhaler triple therapy; PDC: proportion of days covered

The median treatment duration was 102 (98–105) days. At 3, 6, and 12 months from the index date, the PDC by MITT was 81%, 63% and 49%, respectively; this declined to 38% at 24 months (Fig. 4). When the definition of MITT was broadened from concomitant use of all MITT components to any component of MITT, PDC was 74% and 65% at 12 and 24 months, respectively (Fig. 4). Adherence to and persistence with MITT over 24 months are presented in Additional file 1: Fig. S2. The persistent use of MITT declined substantially over time, from 62% at 3 months, to 21% at 12 months, and to 10% at 24 months.

### HRU and costs associated with moderate and severe exacerbation after MITT initiation

Over an observation period of a mean 961 (SD 315) days following the index date, patients with moderate and severe COPD exacerbations had an average of 2.49 (2.46–2.53) visits PPPY. Patients with moderate exacerbations had an average of 1.75 (1.72–1.77) outpatient visits PPPY, and patients with severe exacerbations had an average of 0.77 (0.75–0.78) total (hospitalization, ER, and outpatient) visits PPPY (Table 3). Specifically, patients with severe exacerbations had an average of 0.54 (0.53–0.55) hospitalizations, 0.19 (0.18–0.19) ER visits, and 0.21 (0.20–0.21) outpatient visits PPPY. The mean total healthcare costs associated with moderate and severe COPD exacerbations were 2093 (SD 6010) USD PPPY, comprising mean 173 (SD 451) USD for moderate exacerbations and 1920 (SD 5827) USD for severe COPD exacerbation-related events. The primary driver of healthcare costs associated with severe COPD exacerbations was hospitalization costs (mean 1296 [SD 3494] USD PPPY) (Table 3).

**Table 3:** HRU and costs associated with COPD exacerbations over the observation period (N = 37,400)

	Moderate COPD exacerbations	Severe COPD exacerbations	Total (moderate and severe) COPD exacerbations
*HRU*			
Total visits			
≥ 1 visit, n (%)	N/A	14,731 (39.4)	28,760 (76.9)
Number of visits, mean (SD)	N/A	2.0 (6.2)	6.6 (13.2)
Number of visits, PPPY (95% CI)	N/A	0.77 (0.75–0.78)	2.49 (2.46–2.53)
Outpatient visits			
≥ 1 visit, n (%)	25,850 (69.1)	6944 (18.6)	25,850 (69.1)
Number of visits, mean (SD)	4.6 (10.0)	0.5 (3.0)	5.1 (11.9)
Number of visits, PPPY (95% CI)	1.75 (1.72–1.77)	0.21 (0.20–0.21)	1.95 (1.92–1.98)
ER visits			
≥ 1 visit, n (%)	N/A	8855 (23.7)	8855 (23.7)
Number of visits, mean (SD)	N/A	0.5 (1.9)	0.5 (1.9)
Number of visits, PPPY (95% CI)	N/A	0.19 (0.18–0.19)	0.19 (0.18–0.19)
Hospitalization visits			
≥ 1 visit, n (%)	N/A	14,490 (38.7)	14,490 (38.7)
Number of visits, mean (SD)	N/A	1.4 (3.8)	1.4 (3.8)
Number of visits, PPPY (95% CI)	N/A	0.54 (0.53–0.55)	0.54 (0.53–0.55)
Average duration of hospitalization, days, mean (SD)	N/A	13.4 (10.9)	13.4 (10.9)
*Costs (USD)*			
Total healthcare costs, mean (SD)	463 (1,196)	5125 (15,055)	5588 (13,435)
Medical services costs, mean (SD)	209 (909)	5008 (14,074)	5217 (14,410)
Outpatient costs	209 (909)	101 (3803)	310 (4482)
ER costs	N/A	1445 (4754)	1445 (4754)
Hospitalization costs	N/A	3462 (9,323)	3462 (9323)
Pharmacy costs, mean (SD)	254 (596)	117 (2347)	371 (2493)
Total healthcare costs, PPPY, mean (SD)	173 (451)	1920 (5827)	2093 (6010)
Medical services costs, PPPY, mean (SD)	77 (355)	1882 (5434)	1958 (5568)
Outpatient costs	77 (355)	38 (1517)	115 (1776)
ER costs	N/A	547 (1910)	547 (1910)
Hospitalization costs	N/A	1296 (3494)	1296 (3494)
Pharmacy costs, PPPY, mean (SD)	96 (211)	38 (950)	134 (998)

CI: confidence interval; COPD: chronic obstructive pulmonary disease; ER: emergency room; HRU: healthcare resource utilization; KRW: South Korean Won; N/A: not assessed; PPPM: per person per month; PPPY: per person per year; SD: standard deviation; USD: United States Dollar

The observation period comprised the time from index date until end of data availability; the mean observation period was 960.5 days (Table 2). Moderate exacerbation-related HRU and costs were defined as an HRU event or cost associated with an outpatient visit with a diagnosis of COPD and at least one dispensing for a systemic corticosteroid (intramuscular, intravenous, or oral) or antibiotic within seven days following the encounter. Severe exacerbation-related HRU and costs were defined as an HRU or cost associated with an inpatient hospital stay or ER visit with a diagnosis of COPD. Costs in KRW were indexed to 2016, and converted to USD using the average 2020 exchange rate of 0.0008 USD/KRW. PPPY costs were estimated by multiplying PPPM costs by 12

## Discussion

### Key findings

This study utilized claims data from the South Korea NHI Database to assess the real-world treatment patterns of patients with COPD who were initiated on MITT, as well as the burden of COPD exacerbations. ICS/LABA was the most common maintenance regimen, followed by no maintenance treatment with any of the MITT components, both prior to MITT initiation and after MITT discontinuation; however, the relevance of these findings should be interpreted in the context of the evolving clinical practice. Patients initiated on MITT demonstrated poor adherence and persistence. Patients with COPD in South Korea had a substantial burden of COPD exacerbations at baseline, and across the observation period in the study. In particular, severe COPD exacerbations impose considerable economic burden as they are associated with fewer total visits than moderate COPD exacerbations but incur far greater costs.

### Study population and baseline clinical characteristics

The study population comprised 37,400 patients with moderate-severe COPD, with a mean age of 69 years old. The majority of patients were male and had substantial comorbidities at baseline. These demographics were aligned with previous studies of patients with COPD in South Korea [8, 20]. More than half of patients had ≥ 1 exacerbation during the baseline period with an average of two events per year. This indicates that a considerable proportion of patients in this study met the GOLD 2017 Report’s definition of patients with frequent exacerbations (≥ 2 exacerbations per year), who are anticipated to benefit from triple therapy (i.e., patients who are symptomatic and/or at risk of exacerbation) [9]. Previously published literature has found that delayed initiation of triple therapy was associated with a higher rate of subsequent exacerbations and greater costs associated with inpatient or ER visits [21, 22]. Given the considerable burden of exacerbations at baseline in this study, patients with COPD in South Korea may benefit from earlier optimization of COPD therapy, including earlier initiation of triple therapy.

### Treatment patterns prior to initiation of MITT

It should be noted that the treatment patterns surrounding MITT in this study do not fully reflect the current availability of treatment in South Korea. For instance, LABA/LAMA inhalers were first introduced in 2015–2016 (during the time this study was conducted)–this is reflected in the small proportion of patients (6%) who were on LABA/LAMA prior to initiation of MITT in this study. Similarly, ICS/LABA was the most common treatment that patients received prior to MITT initiation during the baseline period, reflecting the greater availability of these inhalers which became available in South Korea over a decade ago. Following the introduction of LABA/LAMA in South Korea, the COPD treatment landscape is expected to have evolved since this study was conducted. Indeed, the GOLD 2017 Report outlined a preferred initial treatment with LABA/LAMA over ICS/LABA prior to escalation to triple therapy, as LABA/LAMA decreased exacerbations and improved patient-reported outcomes to a greater extent than ICS/LABA in patients with a history of exacerbations [9]. As such, results relating to treatment patterns surrounding MITT in this study should be interpreted in the context of the evolving clinical practice.

While this study may not fully reflect the current treatment patterns surrounding MITT in South Korea, the findings were consistent with other published literature conducted during a similar period. In this study, ICS/LABA was the most common regimen received during the baseline period prior to MITT initiation, followed by no maintenance treatment comprising any of the MITT components. This was also observed globally, where ICS/LABA was found to be the most common treatment before MITT in the UK, Germany, Italy and New Zealand; patients also commonly did not receive any maintenance treatment prior to MITT in France, Germany, Italy and New Zealand [14, 23]. While the latter practice (i.e., initiating MITT for patients who were not on any maintenance treatment comprising any MITT component) is inconsistent with the step-up approach from either LABA/LAMA or ICS/LABA outlined in the GOLD 2017 Report, the tendency to initiate MITT in the first-line setting has been observed by clinical experts in South Korea. This practice may reflect clinical judgment, where patients who present with severe symptoms and/or exacerbation risk may be deemed to benefit from MITT in the first-line setting; indeed, patients in this study had considerable HRU related to hospitalization and ER visits at baseline, suggesting a substantial baseline burden of COPD exacerbations. Similarly, patients may also have been diagnosed at a later and more severe stage, warranting initiation of MITT in the first-line setting, as was observed by Xu et al*.*, who reported that 23% of patients in New Zealand were not on previous therapy prior to MITT initiation, and hypothesized that diagnosis may have been delayed until a late stage before MITT initiation [23]. In addition, the cost barrier for prescription of MITT is low in South Korea due to the low cost of medications or existing reimbursement programs, making the receipt of MITT easily achievable for most of the population.

### Treatment patterns, persistence, and adherence during MITT

In this study, persistence with and adherence to MITT among South Korean patients with COPD were low, with rates of persistent MITT use and adherence declining substantially over time. These findings were consistent with a recently published study on patients with high-grade COPD in South Korea, which reported low adherence to COPD inhalers at year one (35%) through to year four (22%) [24]. Similarly, a real-world study in the US reported mean PDC by MITT at 12 months to be 37% [25], supporting our observation that patients with COPD have poor adherence to and persistence with MITT use. This poor compliance may particularly reflect challenges in complying with multiple-inhaler regimens in general. Indeed, up to 65% of patients with COPD from the US, Germany, and the UK preferred a once-daily single-inhaler regimen over a twice-daily dual-inhaler regimen [26]. Likewise, a US study reported a significantly higher discontinuation rate with multiple-inhaler COPD therapy than single-inhaler COPD therapy [27]. Beyond MITT, patients with COPD have also demonstrated poor adherence to inhaled COPD therapy in general [28]. Patients may therefore benefit from greater emphasis on adherence to inhaled COPD therapy. In a review investigating suboptimal adherence to inhaled medications among patients with COPD and asthma, George et al*.* reported that poor adherence may be related to the medication (e.g., ease of inhaler use, side effects), patients’ choices (e.g., perception that treatment is unnecessary), or are unintentional (e.g., misunderstanding directions or employing the wrong inhaler techniques) [29]. The first factor (relating to the medication) is in line with our findings, where poor adherence to and persistence with MITT and/or complex multiple-inhaler regimens is common among patients with COPD both in this study and globally. At the time the study was conducted, SITT was not available in South Korea, and patients who initiated MITT were thus required to add on or switch inhalers. Such changes in treatment regimen may lead to poor compliance, dissatisfaction, and reduced COPD control [30]. A simplified regimen may be achieved with the use of SITT, which was found to result in greater adherence and persistence, and improved health status and lung function compared with patients who used MITT [11, 31]. Likewise, the factor relating to patients’ choices is consistent with observations by local clinicians that there is a cultural preference, particularly among the elderly in South Korea, for oral medications over inhaled therapy due to familiarity and ease of use [32]. This may also be influenced by the third factor (unintentional factors), where 46–100% of patients with COPD were found to perform incorrect inhaler techniques [33–35], either due to inadequate training or self-modification of the instructed technique [36]. As increased adherence to therapy is known to be associated with improved outcomes, such as a reduced risk of exacerbations [28], increased patient education is important to emphasize the need for adherence to and persistence with inhaled COPD therapy.

### HRU and costs associated with moderate and severe exacerbations after MITT initiation

In this study, substantial HRU and costs were incurred over the observation period following MITT initiation. This considerable burden of COPD exacerbations may be due to the study population already having a high burden of COPD exacerbations at baseline, which may have worsened over the study period, reflecting the natural course of COPD as a progressive disease that worsens over time [37]. In addition, the burden of COPD exacerbations may have been contributed to by poor adherence and persistence (as described above), which has been found to increase the risk of COPD exacerbations [38]. Besides improving adherence, patients may benefit from earlier optimization of COPD therapy, which may be achieved by escalating patients to triple therapy where appropriate. For example, SITT was reported to be associated with significant reductions in moderate or severe exacerbation rates compared with ICS/LABA or LABA/LAMA alone [39, 40]. Earlier optimization of COPD therapy may therefore improve disease control, lowering the risk of exacerbations and associated HRU and costs.

Notably, while severe COPD exacerbations were associated with fewer total visits than moderate COPD exacerbations, severe exacerbations were the main driver of the total costs associated with exacerbations, particularly due to hospitalization costs. This was also consistent with the published literature—in a systematic review of the economic burden associated with COPD, the costs associated with severe COPD exacerbations were consistently greater than those for moderate exacerbations [41]. Treatments that can lower the rate of severe exacerbations are thus expected to lead to substantial cost-savings for the South Korean healthcare system.

### Strengths and limitations

A strength of this study was the use of the NHI claims database, which covers the entire population of South Korea, and contains claims records from all medical facilities. As the loss-to-follow-up was low due to patients remaining in the database until death or migration, we were able to reliably assess a representative population of patients who initiated MITT in South Korea. Secondly, we assessed adherence by calculating PDC, which is the preferred method for measuring adherence in chronic conditions [42]. As such, the use of PDC represents a reliable estimate of adherence in this study. Thirdly, this study included relatively long follow-up periods of up to 24 months, which was useful in assessing changes in persistence, mean PDC and adherence over time, as well as capturing COPD exacerbations over the observation period following MITT initiation. The long follow-up period provided valuable information on the challenges of South Korean patients with COPD in persisting on MITT.

A limitation of this study was that we were unable to assess potentially confounding variables, such as COPD severity or influence of device type, on compliance, as information such as pulmonary function test results or spirometry tests were not recorded in the NHI database. Secondly, while asthma may be a concomitant comorbidity of interest, this study did not report the proportion of patients with asthma. In South Korea, asthma diagnosis codes may be used for reimbursement purposes for an ICS/LABA prescription, and it may be plausible for patients to have asthma diagnosis codes recorded in their claims, without truly having asthma—this practice is known as up-coding [43]. Asthma diagnosis codes may thus be poorly reliable as a reflection of true asthma diagnoses in South Korea, and this study therefore avoided reporting baseline comorbidities based on asthma diagnosis codes. While this limitation may apply to claims data in general [43], local clinicians considered that up-coding affects asthma diagnoses in particular, and is not expected to apply to other diagnosis codes used in this study. Thirdly, as with all studies using claims data where patients’ actual use of medications could not be observed, the claims date may not reflect patients’ actual use of the inhalers upon collection. Relying on claims dates to define MITT use may result in an inaccurate interpretation of patients’ treatment patterns, and adherence and persistence. Finally, while this study was based on claims data that covers the entire population in South Korea, claims data were obtained between 2014 and 2018, and may not be reflective of the current clinical practice in South Korea, especially since SITT is now available.

## Conclusions

Patients with COPD in South Korea experienced frequent COPD exacerbations both at baseline and over the observation period, demonstrating a greater need for earlier optimization of COPD therapy to lower exacerbation risk. Following MITT initiation, adherence to and persistence with MITT was low, suggesting a need for increased emphasis on interventions which may improve adherence to inhaled COPD therapy. Moderate and severe exacerbations incurred substantial costs, and hospitalization for severe exacerbations contributed the most to exacerbation-related costs; hence, treatment alternatives that can reduce the risk of these severe exacerbations are likely to minimize associated healthcare costs.

## Supplementary Information

**Additional file 1**. ** Table S1. **ICD-10-CM codes for Charlson Comorbidities Index-related comorbidities. **Table S2. **ICD-10 codes for Elixhauser comorbidities. **Fig. S1. **Illustration of PDC calculation and sensitivity analysis. **Fig. S2**. Adherence to and persistence with MITT of patients with COPD.

## Data Availability

Anonymized individual participant data and study documents can be requested for further research from www.clinicalstudydatarequest.com.

## Abbreviations

CI: Confidence interval
COPD: Chronic obstructive pulmonary disease
ER: Emergency room
GOLD: Global Initiative for Chronic Obstructive Lung Disease
HRU: Healthcare resource utilization
ICD-10-CM: International Classification of Diseases, 10th Revision, Clinical Modification
ICS: Inhaled corticosteroid
KRW: South Korean Won
LABA: Long-acting beta-agonist
LAMA: Long-acting muscarinic antagonist
NHI: National Health Insurance
MITT: Multiple inhaler triple therapy
PDC: Proportion of days covered
PPPM: Per person per month
PPPY: Per person per year
SD: Standard deviation
SITT: Single inhaler triple therapy
UK: United Kingdom
US: United States
USD: United States dollar
