# Supplementary material for: Treatment patterns and cost of exacerbations in patients with chronic obstructive pulmonary disease using multiple inhaler triple therapy in South Korea
Source: Respir Res. 2022 Sep 5;23:231. doi: 10.1186/s12931-022-02136-0 (PMC9446529; doi:10.1186/s12931-022-02136-0)
Supplement: Supplementary file 1 — Additional file 1. Table S1. ICD-10-CM codes for Charlson Comorbidities Index-related comorbidities. Table S2. ICD-10 codes for Elixhauser comorbidities. Fig. S1. Illustration of PDC calculation and sensitivity analysis. Fig. S2. Adherence to and persistence with MITT of patients with COPD. [file 12931_2022_2136_MOESM1_ESM.docx]

***Additional file***

**Treatment Patterns and Cost of Exacerbations in Patients with Chronic Obstructive Pulmonary Disease Using Multiple Inhaler Triple Therapy in South Korea**

**Authors:** Chang-Hoon Lee,^1^ Mi-Sook Kim,^2^ See-Hwee Yeo,^3^ Chin-Kook Rhee,^4^ Heung-Woo Park,^5–7^ Bo-Ram Yang,^8^ Joongyub Lee,^9^ Eun-Yeong Cho,^10^ Xiaomeng Xu,^3^ Aldo Amador Navarro,^3^ Sumitra Shantakumar,^3^ Dominique Milea,^3^ Nam-Kyong Choi^11^

**Affiliations:** ^1^Division of Pulmonary and Critical Care Medicine, Department of Internal Medicine, Seoul National University Hospital, Seoul, South Korea; ^2^Medical Research Collaborating Center, Seoul National University Hospital, Seoul, South Korea; ^3^Value Evidence & Outcomes, GlaxoSmithKline, Singapore, Singapore; ^4^Division of Pulmonary and Critical Care Medicine, Department of Internal Medicine, Seoul St. Mary’s Hospital, College of Medicine, The Catholic University of Korea, Seoul, South Korea; ^5^Department of Internal Medicine, Seoul National University, Seoul, South Korea; ^6^Institute of Allergy and Clinical Immunology, Seoul National University Medical Research Center, Seoul, South Korea; ^7^Department of Internal Medicine, Seoul National University College of Medicine, Seoul, South Korea; ^8^College of Pharmacy, Chungnam National University, Daejeon, South Korea; ^9^Department of Preventive Medicine, Seoul National University College of Medicine, Seoul, South Korea; ^10^MA Respiratory Department, GlaxoSmithKline, Seoul, South Korea; ^11^Department of Health Convergence, College of Science and Industry Convergence, Ewha Womans University, Seoul, South Korea

**Correspondence to:** Nam-Kyong Choi; Email: [nchoi@ewha.ac.kr](mailto:nchoi@ewha.ac.kr); ORCID: 0000-0003-1153-9928.

**Table S1. ICD-10-CM codes for Charlson Comorbidities Index-related comorbidities**

| Score | Comorbid conditions | ICD-10-CM code |
| --- | --- | --- |
| 1 | Coronary artery disease | I20-I25 |
|  | Congestive heart failure | I11, I50 |
|  | Peripheral vascular disease | I73, I74, I77 |
|  | Cerebrovascular disease | G45, G46, 160-169 |
|  | Dementia | F00-F03, G30 |
|  | Connective tissue disorder | M05-M08, M30-M36 |
|  | Peptic ulcer disease | K25-K8 |
|  | Mild liver disease | B18, K70, K73, K75 |
|  | Diabetes without complications | E10-E14 (0.9) |
|  | Diabetes with end-organ damage | E10-E14 (0.0-0.8) |
|  | Hemiplegia | G81-G82 |
| 2 | Moderate or severe renal disease | All patients |
|  | Tumor without metastases | C00-C76 |
|  | (<5 years) leukemia, lymphoma, multiple myeloma | C81-C96 |
| 3 | Moderate or severe liver disease (cirrhosis) | K72, K74, I85 |
| 6 | Metastatic solid tumor | C77-C80 |
|  | AIDS | B20-B24 |

**Note:** For each decade ≥50 years of age, 1 point was added to the comorbidity score [1].

**Abbreviations:** AIDS: acquired immunodeficiency syndrome; ICD-10-CM: International Classification of Diseases, 10^th^ Revision, Clinical Modification.

**Table S2. ICD-10 codes for Elixhauser comorbidities**

| Comorbidities | ICD-10-CM code |
| --- | --- |
| Congestive heart failure | I09.9, I11.0, I13.0, I13.2, I25.5, I42.0, I42.5–I42.9, I43.x, I50.x, P29.0 |
| Cardiac arrhythmias | I44.1–I44.3, I45.6, I45.9, I47.x–I49.x, R00.0, R00.1, R00.8, T82.1, Z45.0, Z95.0 |
| Valvular disease | A52.0, I05.x–I08.x, I09.1, I09.8, I34.x–I39.x, Q23.0–Q23.3, Z95.2–Z95.4 |
| Pulmonary circulation disorders | I26.x, I27.x, I28.0, I28.8, I28.9 |
| Peripheral vascular disorders | I70.x, I71.x, I73.1, I73.8, I73.9, I77.1, I79.0, I79.2, K55.1, K55.8, K55.9, Z95.8, Z95.9 |
| Hypertension, uncomplicated | I10.x |
| Hypertension, complicated | I11.x–I13.x, I15.x |
| Paralysis | G04.1, G11.4, G80.1, G80.2, G81.x, G82.x, G83.0–G83.4, G83.9 |
| Other neurological disorders | G10.x–G13.x, G20.x–G22.x, G25.4, G25.5, G31.2, G31.8, G31.9, G32.x, G35.x–G37.x, G40.x, G41.x, G93.1, G93.4, R47.0, R56.x |
| Chronic pulmonary disease | I27.8, I27.9, J40.x–J47.x, J60.x–J67.x, J68.4, J70.1, J70.3 |
| Diabetes, uncomplicated | E10.0, E10.1, E10.9, E11.0, E11.1, E11.9, E12.0, E12.1, E12.9, E13.0, E13.1, E13.9, E14.0, E14.1, E14.9 |
| Diabetes, complicated | E10.2–E10.8, E11.2–E11.8, E12.2– E12.8, E13.2–E13.8, E14.2–E14.8 |
| Hypothyroidism | E00.x–E03.x, E89.0 |
| Renal failure | I12.0, I13.1, N18.x, N19.x, N25.0, Z49.0– Z49.2, Z94.0, Z99.2 |
| Liver disease | B18.x, I85.x, I86.4, I98.2, K70.x, K71.1, K71.3–K71.5, K71.7, K72.x–K74.x, K76.0, K76.2–K76.9, Z94.4 |
| Peptic ulcer disease excluding bleeding | K25.7, K25.9, K26.7, K26.9, K27.7, K27.9, K28.7, K28.9 |
| AIDS/HIV | B20.x–B22.x, B24.x |
| Lymphoma | C81.x–C85.x, C88.x, C96.x, C90.0, C90.2 |
| Metastatic cancer | C77.x–C80.x |
| Solid tumor without metastasis | C00.x–C26.x, C30.x–C34.x, C37.x–C41.x, C43.x, C45.x–C58.x, C60.x–C76.x, C97.x |
| Rheumatoid arthritis/ collagen vascular diseases | L94.0, L94.1, L94.3, M05.x, M06.x, M08.x, M12.0, M12.3, M30.x, M31.0–M31.3, M32.x–M35.x, M45.x, M46.1, M46.8, M46.9 |
| Coagulopathy | D65–D68.x, D69.1, D69.3–D69.6 |
| Obesity | E66.x |
| Weight loss | E40.x–E46.x, R63.4, R64 |
| Fluid and electrolyte disorders | E22.2, E86.x, E87.x |
| Blood loss anemia | D50.0 |
| Deficiency anemia | D50.8, D50.9, D51.x–D53.x |
| Alcohol abuse | F10, E52, G62.1, I42.6, K29.2, K70.0, K70.3, K70.9, T51.x, Z50.2, Z71.4, Z72.1 |
| Drug abuse | F11.x–F16.x, F18.x, F19.x, Z71.5, Z72.2 |
| Psychoses | F20.x, F22.x–F25.x, F28.x, F29.x, F30.2, F31.2, F31.5 |
| Depression | F20.4, F31.3–F31.5, F32.x, F33.x, F34.1, F41.2, F43.2 |

**Source:** Quan *et al.* (2005) [2].

**Abbreviations:** AIDS: acquired immunodeficiency syndrome; HIV: human immunodeficiency virus; ICD-10-CM: International Classification of Diseases, 10^th^ Revision, Clinical Modification.

**Fig. S1. Illustration of PDC calculation and sensitivity analysis**

1. **Illustration of PDC calculation**

1. **Illustration of sensitivity analysis for PDC**

**Note:** Each black bar represents a dispensing for a component inhaler of MITT. PDC was defined as the number of days on MITT therapy over a fixed time interval, expressed as a percentage. The sensitivity analysis for PDC (and adherence, defined as PDC by MITT ≥80%) were conducted by broadening the definition of MITT from concomitant use of all MITT components to use of any MITT component.

**Abbreviations:** ICS: inhaled corticosteroid; LABA: long-acting beta agonist; LAMA: long-acting muscarinic antagonist; MITT: multiple inhaler triple therapy; PDC: proportion of days covered.

**Fig. S2. Adherence to and persistence with MITT of patients with COPD**

1. **Adherence of patients with COPD to MITT (N=37,400)**

1. **Persistence of patients with COPD with MITT (N=37,400)**

**Note:** *Total population numbers were 32,743 at 18 months and 26,732 at 24 months. Adherence was defined as PDC by MITT ≥80%. PDC was calculated by dividing the days on therapy for concomitant components or any component of MITT by a fixed time interval, multiplied by 100%. The denominator was 91 days for 3 months, 183 days for 6 months, 365 days for 12 months, 548 days for 18 months, and 730 days for 24 months.

**Abbreviations:** COPD: chronic obstructive pulmonary disease; MITT: multiple inhaler triple therapy; PDC: proportion of days covered.

**References**

1. Chae JW, Song CS, Kim H, et al. Prediction of mortality in patients undergoing maintenance hemodialysis by Charlson Comorbidity Index using ICD-10 database. Nephron Clin Pract. 2011;117:c379-384.

2. Quan H, Sundararajan V, Halfon P, et al. Coding algorithms for defining comorbidities in ICD-9-CM and ICD-10 administrative data. Med Care. 2005;43:1130–9.
